# *Panax Notoginseng* Protects against Diabetes-Associated Endothelial Dysfunction: Comparison between Ethanolic Extract and Total Saponin

**DOI:** 10.1155/2021/4722797

**Published:** 2021-09-04

**Authors:** Xutao Zhang, Chunxiu Zhou, Lingchao Miao, Yi Tan, Yan Zhou, Meng Sam Cheong, Yu Huang, Yitao Wang, Hua Yu, Wai San Cheang

**Affiliations:** ^1^State Key Laboratory of Quality Research in Chinese Medicine, Institute of Chinese Medical Sciences, University of Macau, Macao SAR, China; ^2^School of Biomedical Sciences, Chinese University of Hong Kong, Hong Kong SAR, China

## Abstract

Previous studies revealed a cardioprotective potential of *Panax notoginseng* to relieve acute myocardial infarction and focal cerebral ischemia-reperfusion. However, whether *P. notoginseng* protects endothelial function in diabetes and the underlying mechanisms remain to be explored. *P. notoginseng* contains several chemical components including saponins, which are commonly believed as the major bioactive ingredients. The present study was aimed to examine and compare the vaso-protective effects of the ethanolic extract of *P. notoginseng* (PNE) and total saponin (PNS). Both aortas and carotid arteries were isolated from male C57BL/6J mice for *ex vivo* treatment with risk factors (high glucose or tunicamycin) with and without the presence of PNS and PNE. Diabetic model was established by feeding the mice with a high-fat diet (45% kcal% fat) for 12 weeks, while PNS and PNE were administrated by oral gavage at 20 mg/kg/day for another 4 weeks. *Ex vivo* exposure to high glucose impaired acetylcholine-induced endothelium-dependent relaxations in mouse aortas, decreased phosphorylation of AMPK and eNOS, and induced endoplasmic reticulum (ER) stress and oxidative stress. These effects were reversed by cotreatment of PNS and PNE with PNS being more potent. Furthermore, the vaso-protective effects were abolished by Compound C (AMPK inhibitor). Chronic treatment with PNS and PNE improved endothelium-dependent relaxations and alleviated ER stress and oxidative stress in aortas from high-fat diet-induced obese mice. PNE was more effective to improve glucose sensitivity and normalize blood pressure in diabetic mice. The present results showed that PNS and PNE reduced ER stress and oxidative stress and, subsequently, improved endothelial function in diabetes through AMPK activation. This study provides new inspiration on the therapeutic potential of *P. notoginseng* extract against vascular diseases associated with metabolic disorders.

## 1. Introduction

*Panax notoginseng* (Burk) F.H. Chen is a widely used medicinal plant in East Asian countries for thousands of years. Radix Notoginseng, also named as Sanqi or Tianqi in Chinese, is the dried root of *P. notoginseng*, which is the main part used for medical purpose, while the leaves, fruits, and flowers possess medicinal value [1]. According to the Chinese Pharmacopoeia, its traditional pharmacological effect is to disperse blood stasis and stop bleeding, reduce swelling and relieve pain. Increasing pharmacological and clinical evidence have supported that *P. notoginseng*, particularly its saponin components, possess multiple beneficial effects. *P. notoginseng* saponins (PNS) and ginsenosides Rb2 and Rd2 inhibit platelet activity [2–4]. PNS exhibit antiobesity [5] and antidiabetic properties [6] and also attenuate nonalcoholic fatty liver disease [7]. Of note, *P. notoginseng* is found to be cardio-protective as PNS protect against acute myocardial infarction in mice [8] and relieve the focal cerebral ischemia-reperfusion in rats [9], while notoginsenoside R1 promotes angiogenic activity in endothelial cells and zebrafish [10]. Furthermore, notoginsenoside R1 was reported to prevent cardiomyocyte apoptosis and ischemic/reperfusion injuries via inhibition of signaling pathways related to oxidative stress and endoplasmic reticulum (ER) stress [11]. Another recent study showed that PNS protect against thapsigargin-induced mitochondrial injury, reducing ROS accumulation and Ca^2+^-dependent ER stress [12].

ER is an important organelle for the synthesis and folding of membrane and secretory proteins, but disruption of ER function leading to ER stress is a critical contributor to cellular dysfunction and metabolic disorder and related complications [13–15]. Complex signaling cascades are activated in response to ER stress, initiated by phosphorylation of three ER membrane-associated proteins: PKR-like endoplasmic reticulum kinase (PERK), inositol requiring enzyme 1 (IRE1), and activating transcription factor 6 (ATF6) [16]. A crosstalk is suggested between ER stress and oxidative stress in cardiovascular diseases [17]. The production of reactive oxygen species (ROS) is elevated during ER stress via NADPH oxidases, mainly Nox2 and Nox4 [18]. On the other hand, ROS accumulation further promotes ER stress [19], where mitochondrial ROS plays an important role [20]. In consistence, our previous findings support that ER stress induces ROS production and endothelial dysfunction; and that alleviation of ER stress and oxidative stress protects against diabetic vasculopathy [21, 22].

5′-Adenosine monophosphate-activated protein kinase (AMPK) plays a key role both in regulating metabolic homeostasis and cardiovascular biology; its phosphorylation contributes to the activation of endothelial nitric oxide synthase (eNOS) [23]. AMPK activation was shown to mitigate ER stress and oxidative and rescue vascular function in diabetes [24, 25].

Previous studies showed that *P. notoginseng*, in particular PNS, improves vascular and metabolic functions. However, little is known whether *P. notoginseng* protects endothelial function in diabetes and the underlying mechanisms. Importantly, most studies showed PNS as the major bioactive components of *P. notoginseng*. Herein, we examined and compared the vaso-protective effects of total ethanolic extract of *P. notoginseng* (PNE) and PNS in alleviating ER stress and endothelial dysfunction associated with diabetes *in vitro* and *in vivo.*

## 2. Materials and Methods

### 2.1. Preparation of Herbal Extract

The air-dried and powered *Panax notoginseng* powder (Sanqi or Tianqi) was extracted with 95% ethanol (1 : 10 *w*/*v*) and refluxed for 1 h twice. The filtered extracts were combined and concentrated under rotate reduced pressure to remove ethanol. The concentrated extract was then lyophilized with a Virtis Freeze Dryer (Te Virtis Company, USA) to obtain the final freeze-dried powder (PNE). PNS was purchased from Yunnan Yunke Pharmaceutical Co. Ltd (China).

### 2.2. UPLC Analysis

The chemical constitutions of PNS and PNE were quantitatively determined using a Waters ACQUITY-UPLC CLASS system (Waters Corp., USA) coupled with an ACQUITY UPLC BEH phenyl column (150 mm × 2.1 mm, 1.7 *μ*m) maintained at 45°C. Elution was performed with a mobile phase of water (A) and acetonitrile (B) under a gradient program: 0-10 min, 19% B; 10-15 min, 19-35% B; 15-20 min, 35-38% B. The flow rate was 0.4 mL/min, and the injection volume was 2 *μ*L. The analytes were monitored at the UV wavelength of 205 nm. Prior to the next injection, the column was washed with 100% B for 2 min and then equilibrated with the initial mobile phase for 3 min. Notogisenoside R1, ginsenoside Rb1, ginsenoside Re, ginsenoside Rg1, and ginsenoside Rd (the purities of all standards were higher than 98% by HPLC analysis) were purchased from Chengdu Pufei De Biotech Co., Ltd. (China). Acetonitrile was purchased from RCI Labscan Limited (Thailand) of HPLC grade. Milli-Q water was prepared using a Milli-Q system (Millipore, USA).

### 2.3. Animals and Drug Treatments

The use of mice and research protocol was approved by the Animal Research Ethics Committee, University of Macau, and conformed to the National Institutes of Health guidelines for the Care of Use of Laboratory Animals. Male C57BL/6J mice were purchased from the Faculty of Healthy Science Animal Centre of the University of Macau and housed in a temperature-controlled room (22-24°C) with a 12-hr light/dark cycle. The mice at the age of 6 weeks were fed with a high-fat diet (45% kcal% fat) for 12 weeks to establish a type 2 diabetic model followed by oral administration of vehicle (water), PNS, or PNE at 20 mg/kg body weight daily for another 4 weeks. Mice fed with a normal chow diet served as control.

### 2.4. Blood Pressure Measurement

Systolic (SBP) and diastolic (DBP) blood pressure were measured by the CODA noninvasive blood pressure system (a tail-cuff method, Kent Scientific Corporation, USA) in conscious mice.

### 2.5. Blood Glucose Measurement

After 6-h fasting, blood was drawn from the mouse tail to determine fasting blood glucose (FBG) level using a commercial glucometer, and mice with FBG >11 mM were considered as diabetic. For oral glucose tolerance test (OGTT), mice after 6-h fasting were loaded with glucose solution (1.2 g/kg body weight) by oral gavage and blood glucose levels were measured at 0, 15, 30, 60, and 120 min. For the insulin tolerance test (ITT), mice after 2-h fasting were injected with insulin (0.5 U/kg body weight) intraperitoneal, and the blood glucose levels were detected at the same time intervals as OGTT.

### 2.6. *Ex Vivo* Culture of Mouse Aortas

After mice were sacrificed, both thoracic aortas and carotid arteries were rapidly removed and carefully dissected free from adjacent connective tissues in sterile phosphate-buffered saline (PBS). Arteries were cut into segments (~2 mm in length) and were incubated in a Dulbecco's Modified Eagle's Media (DMEM, Gibco, USA) supplemented with 10% fetal bovine serum (FBS, Gibco), plus 100 IU/mL penicillin and 100 *μ*g/mL streptomycin. Arterial segments were incubated with normal medium with mannitol added as osmotic control, high glucose (30 mM) medium or high glucose medium plus individual drugs including different concentrations of PNE or PNS and Compound C (5 *μ*M, AMPK inhibitor, Sigma-Aldrich) in incubator at 37°C for 48 h. The ring segments were then collected for functional studies in wire myograph, Western blotting, and fluorescence imaging.

### 2.7. Isometric Force Measurement in Wire Myograph

Aortic segments (~2 mm) were suspended in a Multi Myograph System (Danish Myo Technology, Denmark) to determine the changes in isometric tension. Mouse aortas were stretched to an optimal baseline tension of 3 mN with equilibration for 60 min before they were contracted by 60 mM KCl. Endothelium-dependent relaxations (EDRs) were studied in phenylephrine (Phe, 3 *μ*M, Sigma-Aldrich) precontracted endothelium-intact rings in response to acetylcholine (ACh, 3 nM-10 *μ*M, Sigma-Aldrich). Endothelium-independent relaxations were measured in response to sodium nitroprusside (SNP, 1 nM-10 *μ*M, Sigma-Aldrich). Each experiment was performed on rings prepared from different mice.

### 2.8. Western Blot

Aortas snap frozen in liquid nitrogen were subsequently homogenized in ice-cold RIPA lysis buffer. The homogenates were incubated on ice for 30 min and then centrifuged for 20 min at 20,000 g. The supernatant was collected and protein concentration was measured using BCA assay. Protein sample (15 *μ*g) was electrophoresed through 7.5%-12.5% (SDS-PAGE) and transferred to the PVDF membrane (Millipore) using wet transfer (BIO-RAD, USA). Nonspecific binding sites were blocked by 5% nonfat milk or 1% BSA in 0.05% Tween-20 PBS, and the membranes were then probed with the primary antibodies against target proteins at 4°C overnight; followed by incubation with corresponding HRP-conjugated secondary antibodies. The membranes were visualized with an American ECLTM Advanced Western Blotting Detection Kit (GE Healthcare Life Sciences, Sweden) and scanned by ChemiDoc MP Imaging System (BIO-RAD). GAPDH was selected as a housekeeping protein for checking equal loading of each sample.

### 2.9. Culture of Human Umbilical Cord Vein Endothelial Cells (HUVECs)

HUVECs (Lonza) were grown in EGM supplemented with Bulletkit (Lonza) in 75 cm^2^ flasks and maintained at 37°C in a humidified atmosphere of 5% CO_2_. Experiments were performed on cells at passage 4-8 to treat with high glucose, PNE, PNS, or Compound C when 80-90% confluency was achieved.

### 2.10. Detection of ROS by Dihydroethidium (DHE) Staining

Segments of or aortas (chronic treatment) and carotid arteries (*ex vivo* treatment) were frozen in OCT compound (Tissue-Tek) and sliced into sections (10 *μ*m) using a Leica CM 1000 cryostat. The arterial sections and treated HUVECs were incubated in DHE- (5 *μ*M; Invitrogen) containing normal physiological saline solution (NPSS) at 37°C for 15 min. NPSS contained (mM): 140 NaCl, 5 KCl, 1 CaCl_2_, 1 MgCl_2_, 10 glucose, and 5 HEPES (pH 7.4). Fluorescence images were obtained with the Leica TCS SP8 Confocal Laser Scanning Microscope System (Leica Microsystems, Germany) by measuring the fluorescence intensity at excitation 515 nm and emission 585 nm.

### 2.11. Statistical Analysis

Data were expressed as mean ± SEM of *n*-independent experiments. Comparisons were analyzed using one-way ANOVA and *t*-test in the GraphPad Prism software (GraphPad Software, USA). *P* < 0.05 was regarded to be a statistically significant difference.

## 3. Results

### 3.1. Quantitative Analysis of Ethanolic Extract (PNE) and Total Saponin of *Panax Notoginseng* (PNS)

**Figure 1:**
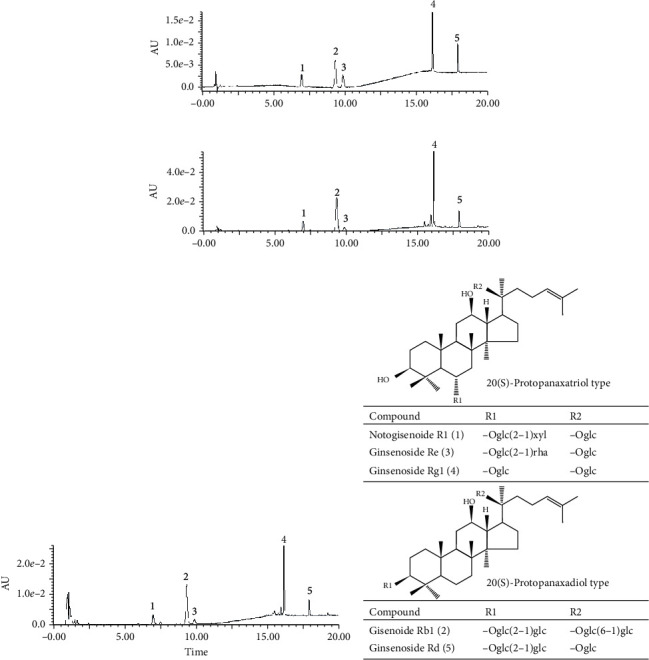
Quantitative analysis of *Panax notoginseng* ethanolic extract (PNE) and total saponins (PNS). Chromatograms of (a) mixed standards, (b) PNS, and (c) PNE by UPLC analysis. 1: Notogisenoside R1; 2: Ginsenoside Rb1; 3: Ginsenoside Re; 4: Ginsenoside Rg1; 5: Ginsenoside Rd. (d) Chemical structures of notoginsenoside R1, ginsenosides Rg1, Re, Rb1, and Rd. Glc, *β*-D-glucose; Rha, *α*-L-rhamnose; Xyl, *β*-D-xylose.

The contents of notogisenoside R1, ginsenoside Rb1, ginsenoside Re, ginsenoside Rg1, and ginsenoside Rd in PNS and PNE were determined using UPLC analysis. The chromatograms of mixed standards, PNS (0.5 mg/mL) and PNE (0.5 mg/mL), were illustrated in Figure 1. All compounds were chromatographically separated with the developed analytical method. The contents of five saponins in PNS and PNE were shown in Table 1.

### 3.2. PNS and PNE Improve Vascular Functions in Hyperglycemic Condition

**Figure 2:**
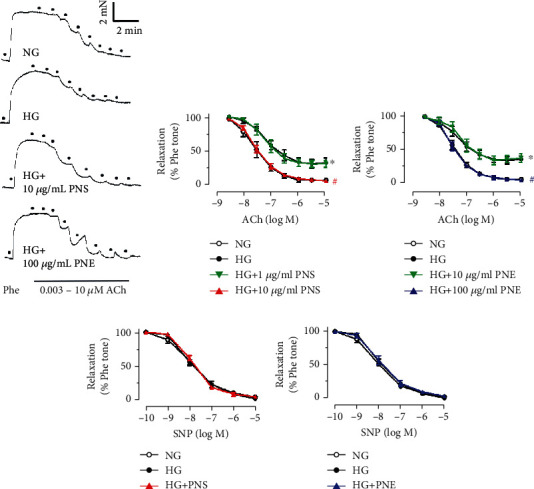
*Panax notoginseng* extract protects endothelial function in a hyperglycemic condition. (a) Representative traces and (b, c) summarized data showing the effects of PNS and PNE on acetylcholine- (ACh-) induced EDRs in mouse aortas exposed to high glucose (HG, 30 mM, 48 h). (d, e) Sodium nitroprusside- (SNP-) induced endothelium-independent relaxations in aortas. Data are mean ± SEM of 4 experiments. ^∗^*P* < 0.05 vs. normal glucose (NG, 5.55 mM with mannitol); ^#^*P* < 0.05 vs. HG.

Aortic rings from C57BL/6J mice were cultured with high glucose (30 mM, 48 h) to mimic the hyperglycemic condition in diabetes. High glucose-induced impairment of EDRs in response to ACh was improved by PNS and PNE (Figures 2(a)–2(c)). PNS exhibited a more potent vaso-protective effect than PNE *ex vivo*. PNS had no effect at 1 *μ*g/mL but was effective at 10 *μ*g/mL to restore EDRs comparable to the control (5.55 mM glucose in DMEM with the addition of mannitol as osmotic control). By contrast, PNE ameliorated high glucose-induced endothelial dysfunction at a higher concentration of 100 *μ*g/mL. Endothelium-independent relaxations to SNP (Figures 2(d) and 2(e)) were not altered among groups, indicating the response of vascular smooth muscle to NO was normal. The lowest effective concentrations of PNS and PNE observed in the functional studies were chosen for further experiments to study the underlying mechanisms: 10 *μ*g/mL and 100 *μ*g/mL, respectively.

### 3.3. PNS and PNE Enhance AMPK/eNOS Pathway and Attenuate ER Stress

**Figure 3:**
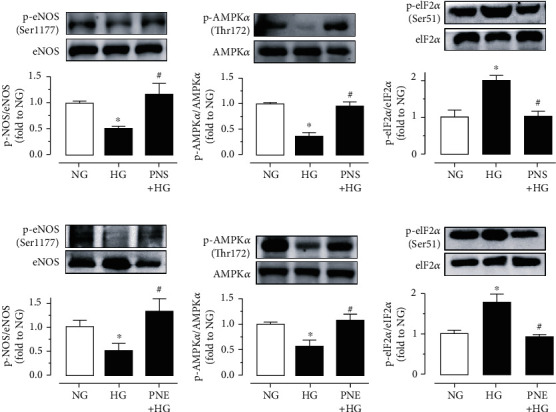
PNS and PNE stimulate AMPK*α*/eNOS and reduce ER stress. Western blotting showing (a) the phosphorylation of eNOS at Ser1177 (p-eNOS; 140 kDa), (b) phosphorylation of AMPK*α* at Thr172 (p-AMPK*α*; 62 kDa), and (c) phosphorylation of eIF2*α* at Ser52 (p-eIF2*α*; 36 kDa) compared to their corresponding total protein in mouse aortas treated with high glucose (HG, 30 mM) and PNS (10 *μ*g/mL) for 48 h. (d–f) Protein expressions in mouse aortas treated with PNE (100 *μ*g/mL). Data are mean ± SEM of 4 experiments. ^∗^*P* < 0.05 vs. normal glucose (NG, 5.5 mM with mannitol); ^#^*P* < 0.05 vs. HG.

**Figure 4:**
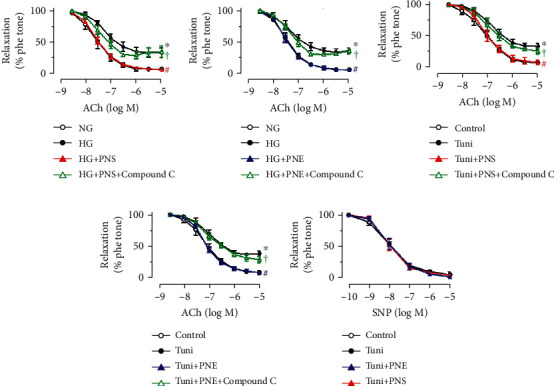
AMPK and ER stress modulates the beneficial effect of PNS and PNE on endothelial function. (a, b) Effect of AMPK inhibitor Compound C (5 *μ*M) on ACh-induced EDRs in mouse aortas treated with high glucose (HG, 30 mM), PNS (10 *μ*g/mL) and PNE (100 *μ*g/mL) for 48 h. Effect of pretreatment with ER stress inducer tunicamycin (Tuni, 2 *μ*g/mL, 24 h) and cotreatment with PNS (10 *μ*g/mL) or PNE (100 *μ*g/mL) on (C and D) ACh-induced and (e) SNP-induced relaxations in mouse aortas. Data are mean ± SEM of 4 experiments. ^∗^*P* < 0.05 vs. normal glucose (NG, 5.5 mM with mannitol) or control; ^#^*P* < 0.05 vs. HG or Tuni; ^†^*P* < 0.05 vs. HG+PNS, HG+PNE, Tuni+PNS, or Tuni+PNE.

Upon treatment with high glucose for 48 h, the phosphorylation of eNOS at Ser1177 and phosphorylation of AMPK*α* at Thr172 was suppressed, while phosphorylated eukaryotic initiation factor 2*α* (eIF2*α*) at Ser52 was elevated in mouse aortas (Figure 3). Such changes were reversed by co-treatment with PNS (10 *μ*g/mL) (Figures 3(a)–3(c)) and PNE (100 *μ*g/mL) (Figures 3(d)–3(f)). In addition, the vaso-protective effect of PNS and PNE against high glucose-induced impairment of EDRs was abolished by AMPK inhibitor Compound C (5 *μ*M) (Figures 4(a) and 4(b)). *Ex vivo* 24-h exposure of mouse aortas to the ER stress inducer tunicamycin (2 *μ*g/mL) reduced EDRs, which were restored by coincubation with PNS (10 *μ*g/mL) (Figure 4(c)) and PNE (100 *μ*g/mL) (Figure 4(d)) without affecting SNP-induced endothelium-independent relaxations (Figure 4(e)). Similarly, the improvements in EDRs were inhibited by Compound C.

### 3.4. PNS and PNE Suppress Oxidative Stress through an AMPK-Dependent Mechanism

**Figure 5:**
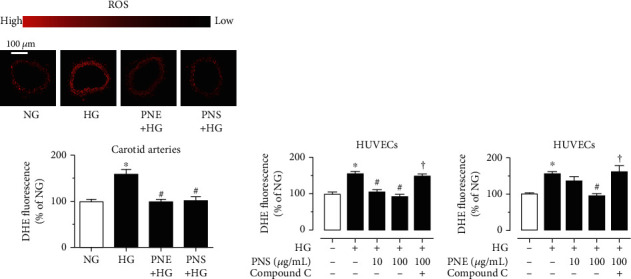
PNS and PNE attenuate oxidative stress. (a) Representative images and summarized graph showing DHE intensity (oxidative stress indicator) in mouse carotid arteries treated with high glucose (30 mM, 4 h), PNS (10 *μ*g/mL), and PNE (100 *μ*g/mL). (b, c) Inhibitive effects of PNS and PNE on high glucose-induced oxidative stress in HUVECs. Data are mean ± SEM of 4 experiments. ^∗^*P* < 0.05 vs. normal glucose (NG, 5.5 mM with mannitol); ^#^*P* < 0.05 vs. HG.

Both PNS (10 *μ*g/mL) and PNE (100 *μ*g/mL) effectively inhibited high glucose- (30 mM, 4 h) induced ROS elevation in mouse carotid arteries (Figure 5(a)) and in HUVECs (Figures 5(b) and 5(c)). PNS was more potent to reduce oxidative stress at 10 *μ*g/mL, while PNE was only effective at 100 *μ*g/mL. The antioxidative effects of both PNS and PNE were reversed by Compound C (5 *μ*M), indicating that AMPK activation contributes to the antioxidative activity of PNS and PNE.

### 3.5. Chronic Treatment with PNS and PNE Ameliorates Endothelial Dysfunction and Oxidative Stress in Diet-Induced Obese Mice

**Figure 6:**
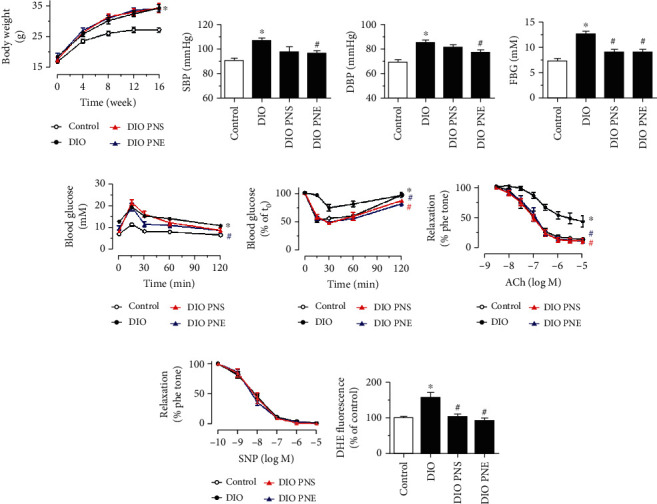
Vaso-protective effect of PNS and PNE in diet-induced obese (DIO) mice. (a) Changes in body weight in mice fed with high-fat or normal chow diet for 16 weeks and administered with vehicle (water), PNS, or PNE at 20 mg/kg body weight daily by oral gavage at the last 4 weeks. (b, c) Changes in systolic (SBP) and diastolic (DBP) blood pressure measured by tail-cuff method. (d) Changes in fast blood glucose (FBG) measured after fasting the mice for 6 h. (e) Oral glucose tolerance test (OGTT) upon 6-h fasting. (f) Insulin tolerance test (ITT) upon 2-h fasting. (g) Effect of oral administration of PNS or PNE (20 mg/kg/day, 4 weeks) on ACh-induced relaxations in aortas from DIO mice. (h) SNP-induced endothelium-independent relaxations in mouse aortas. (i) Changes in DHE intensity (oxidative stress indicator) in mouse aortas. Data are mean ± SEM of 5 experiments. ^∗^*P* < 0.05 vs. control; ^#^*P* < 0.05 vs. DIO.

To examine the vascular benefit of PNS and PNE treatments *in vivo*, diet-induced obese (DIO) mice were orally administered with PNS and PNE at 20 mg/kg/day for 4 weeks. Feeding with a high-fat diet for 16 weeks markedly increased body weight as compared to the control mice feeding with normal chow whereas PNS and PNE treatments exerted no effect on body weight (Figure 6(a)). In contrast to the *in vitro* data, chronic PNE treatment showed more effective protection of endothelial function and glycemic control than PNS. Four-week PNE treatment normalized the elevated systolic and diastolic blood pressures in DIO mice, while PNS exhibited a small but insignificant effect (Figures 6(b) and 6(c)). Fasting glucose levels of DIO mice were higher than those of control and were greater than 11 mM, indicating the success of establishing a diabetic model in mice (Figure 6(d)). Oral glucose (Figure 6(e)) and insulin tolerance tests (Figure 6(f)) revealed impaired glucose sensitivity and insulin irresponsiveness in DIO mice comparing to lean nondiabetic control mice. Both PNS and PNE treatments normalized the elevated fasting glucose level and improved insulin sensitivity; nevertheless, the reduced glucose tolerance was moderately reversed by PNE treatment but was insensitive to PNS treatment. ACh-induced EDRs were decreased in DIO mice and chronic PNS and PNE treatments restored the impaired EDRs (Figure 6(g)) without affecting SNP-induced endothelium-independent relaxations in aortas (Figure 6(h)). The elevated ROS level was also reversed by PNS and PNE treatments in aortas from DIO mice (Figure 6(i)).

### 3.6. PNS and PNE Activate AMPK/eNOS and Alleviate ER Stress in Aortas of DIO Mice

**Figure 7:**
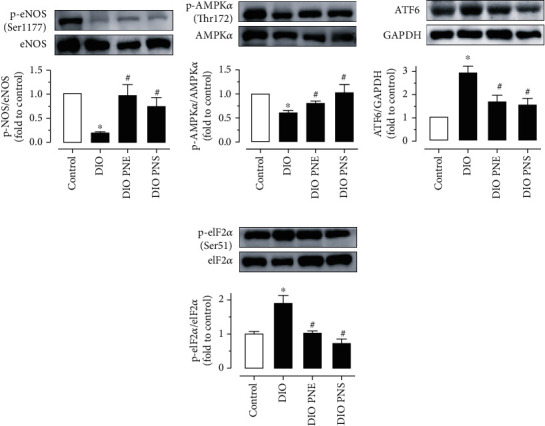
Chronic PNS and PNE treatments activate AMPK/eNOS pathway and reduce ER stress. Western blot data showing (a) the phosphorylation at Ser1177 and total eNOS (140 kDa), (b) phosphorylation at Thr172 and total AMPK*α* (62 kDa), (c) cleaved ATF6 (50 kDa) compared to GAPDH, and (d) phosphorylation at Ser52 and total eIF2*α* (36 kDa) in aortas from four groups of mice. Data are mean ± SEM of 5 experiments. ^∗^*P* < 0.05 vs. control; ^#^*P* < 0.05 vs. DIO.

In aortas from DIO mice, eNOS phosphorylation at Ser1177 and AMPK*α* phosphorylation at Thr172 were downregulated (Figures 7(a) and 7(b)), while ER stress markers including cleaved activating transcription factor 6 (ATF6) and eIF2*α* phosphorylation at Ser52 were upregulated compared with lean control (Figures 7(c) and 7(d)). PNS and PNE treatments reversed these changes on AMPK/eNOS and ER stress in DIO mice.

## 4. Discussion

The present study used multiple approaches to elucidate and compare the effects of PNS and PNE on endothelial dysfunction in diet-induced obese mice. Treatment with either PNS or PNE rescued the impaired endothelial function in a hyperglycemic condition and in diabetic obese mice, accompanied by enhancement of vascular AMPK/eNOS activities with inhibition of ER stress and oxidative stress. PNS was found to be more potent *ex vivo* and *in vitro* but PNE was more effective *in vivo*.

With the advances of scientific development and emergence of new extraction or identification methods, an increasing number of novel compounds are being discovered from *P. notoginseng*. The chemical composition varies in different parts of *P. notoginseng* and is affected by the processing method. Until now, more than 200 phytochemicals have been isolated and identified from various parts of *P. notoginseng*, including saponins, polysaccharides, dencichine (a nonprotein amino acid), flavonoids, fatty acids, and volatile oil [1, 26]. According to the data of UPLC analysis, PNS used in the present study mainly contains five saponins including notogisenoside R1 and ginsenosides Rb1, Re, Rg1, and Rd, while the five saponins account for around half of the contents in PNE. Another half of PNE probably consists of other chemical components such as polysaccharides, dencichine, flavonoids, fatty acids, and volatile oil, which needs to be identified by further investigation.

AMPK activation stimulates eNOS and increases NO bioavailability [23]. Moreover, AMPK has been widely confirmed to decrease ROS production in diabetes-related diseases [27–29]. Previous studies showed that AMPK activation suppresses ER stress and control of ER stress is pivotal for vascular function [24, 25, 30]. PNS was described to promote angiogenesis in HUVECs [31] and protect against acute myocardial infarction in mice [8] through AMPK activation. Our observations are in line with these previous studies, reporting that *ex vivo* high glucose exposure impaired EDRs and stimulated ROS production, which were reversed by cotreatment of PNS and PNE. Importantly, these protective effects of PNS and PNE were absent when AMPK inhibitor Compound C was added. PNS and PNE suppressed the phosphorylation of eIF2*α*, one of the ER stress markers triggered by high glucose exposure. Likewise, induction of ER stress by tunicamycin impaired EDRs in conduit aortas which were rescued by PNS and PNE, and such improvement was abolished in the presence of Compound C. The present results show that both PNS and PNE effectively improved high glucose-induced endothelial dysfunction with alleviation of ER stress and oxidative stress through the AMPK/eNOS pathway. Besides, PNS was more potent to exert vaso-protective effects at 10 *μ*g/mL but PNE showed no improvement at the same concentration.

Excessive nutrient such as glucose in diabetes is one of the common risk factors causing vascular complications [32]. Chronic treatment with PNS and PNE did not affect body weight but improved glucose metabolism, which might partially contribute to the *in vivo* beneficial effects to improve EDRs and normalize blood pressure in diabetic and obese mice. The *in vivo* study showed contradictory findings with the *ex vivo* and *in vitro* studies. Treatment of DIO mice with the same amount of PNS and PNE (20 mg/kg/day for 4 weeks) potently improved EDRs and reduced ROS levels to the similar degree. However, PNE but not PNS significantly reduced systolic and diastolic blood pressure. This may be explained by the difference of effects on glucose metabolism for these two extracts: PNE moderately improved glucose sensitivity but PNS did not. The *ex vivo* experiments showed treatment of mouse aortas with tunicamycin impaired EDRs, which were improved by PNS and PNE, where ambient glucose was constant. Therefore, these results strongly suggest that PNS and PNE have direct vaso-protective effects through alleviation of ER stress and oxidative stress even without affecting glucose metabolism. Western blot data showed that chronic treatment with PNS and PNE resulted in increased phosphorylation of eNOS and AMPK as well as decreased levels of both ER stress and oxidative stress, indicating that increased AMPK/eNOS activity and suppressed ER stress are likely to mediate the vaso-protective effects of PNS and PNE.

PNS is commonly considered as the major pharmacological active ingredients of *P. notoginseng*. However, our present study demonstrates that the whole ethanolic extract containing both saponins and other chemical compounds exhibited better beneficial effects in improving glucose metabolism, reversing hypertensive condition, and protecting endothelial function. Vascular function is regulated by multiple factors including glucose metabolism [33, 34]. The additional benefits of PNE may be attributed to the presence of other active ingredients apart from saponins, or saponins may interact with other ingredients to have synergistic effects in improving vascular function in diabetes. This possibility remains to be further explored.

## 5. Conclusions

The present results show that PNS and PNE protected against diabetes-associated endothelial dysfunction through activating the AMPK/eNOS pathway, accompanied by alleviation of ER stress and oxidative stress. The current novel findings support the therapeutic potential of *P. notoginseng* in combating vascular and metabolic diseases.

## Figures and Tables

**Table 1:** Contents (%, *g*/*g*) of five main saponins in PNS and PNE.

Compound	PNS (%, *g*/*g*)	PNE (%, *g*/*g*)
Notogisenoside R1	11.20 ± 0.32	5.45 ± 0.25
Ginsenoside Rb1	33.51 ± 0.63	18.96 ± 0.52
Ginsenoside Re	4.16 ± 0.01	2.18 ± 0.06
Ginsenoside Rg1	40.57 ± 0.69	17.71 ± 0.58
Ginsenoside Rd	8.13 ± 0.14	4.17 ± 0.16
Total	97.57	48.47

## Data Availability

The data used to support the content of this manuscript are available upon request to the corresponding author.
